# Ruptured abdominal aortic aneurysm in 14-year-old female with tuberous sclerosis

**DOI:** 10.1016/j.jvscit.2026.102328

**Published:** 2026-05-22

**Authors:** Austin Clements, Sellers Boudreau, Benjamin Pearce

**Affiliations:** Department of Vascular Surgery, The University of Alabama, Birmingham, AL

## Abstract

Abdominal aortic aneurysms (AAAs) in pediatric populations with tuberous sclerosis complex (TSC) are limited to sporadic case reports and small series within the distant literature. TSC is a multisystem autosomal-dominant genetic disorder with variable penetrance characterized by benign tumors and dysplastic lesions in multiple organs. Although not well understood, there remains a critical association of TSC with pediatric AAAs. We herein describe a case report and operative outcome of a 14-year-old girl with a history of TSC who presented to the emergency department with a ruptured infrarenal AAA.

Abdominal aortic aneurysms (AAAs) are rare vascular entities within the pediatric population. Currently, only 12 case reports document the development of an AAA in pediatric patients. Although documented, the exact association of AAAs and tuberous sclerosis complex (TSC) remains poorly understood. TSC is an autosomal-dominant multisystem disorder caused by deranged variants in the *TSC1* or *TSC2* genes with variable penetrance, leading to dysregulated mammalian target of rapamycin signaling and hamartomatous lesions across organ systems. Phenotypic expression of TSC portends seizures, intellectual disability, facial adenoma sebaceum, and visceral or cutaneous hamartomas.^1^ The majority of pediatric AAAs are associated with systemic disorders such as Ehlers-Danlos syndrome, Takayasu arteritis, and Kawasaki disease.^2^ Herein, we report the case of a 14-year-old girl with TSC presenting with a ruptured infrarenal AAA (I-AAA).

## Case report

A 14-year-old girl presented to the emergency department with acute abdominal pain and peritonitis. Notably, she has a personal history of TSC and seizures. She has a family history of TSC with multiple siblings and father diagnosed with the condition; however, they were not afflicted with an arterial pathology. On physical examination, there were multiple hyperpigmented macules, ash leaf spots, fascial fibromas, and extremity fibromas. The abdomen was diffusely tender with focal peritonitis along the midline with an obvious pulsatile mass. Vital signs showed tachycardia to 120 beats per minute, hypertension (180/90 mm Hg), and adequate oxygenation on room air. Her vascular examination was intact with bilateral palpable femoral, radial, and pedal pulses. Axial and coronal computed tomography angiography demonstrated an isolated 7.2-cm retroperitoneally contained a ruptured I-AAA (Fig 1, Fig 2, Fig 3). Impulse control was promptly initiated to lessen further exsanguination risk and the patient was transported emergently to the operating room for open repair of the ruptured I-AAA.

**Fig 1:**
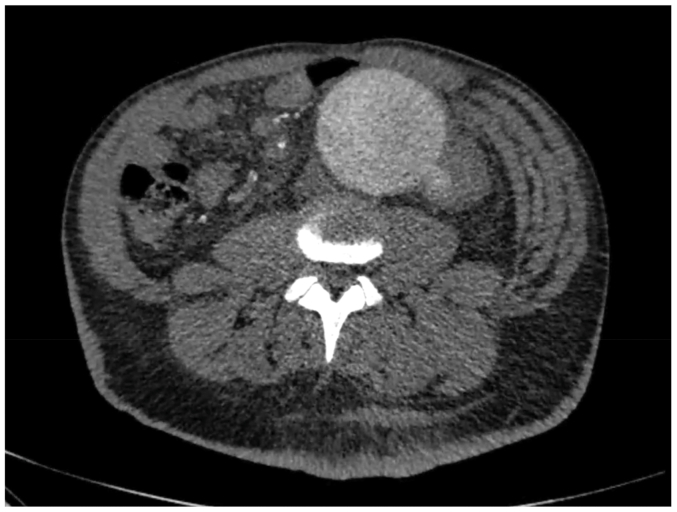
Computed tomography angiography of the ruptured abdominal aortic aneurysm (AAA) with active retroperitoneal extravasation (axial view).

**Fig 2:**
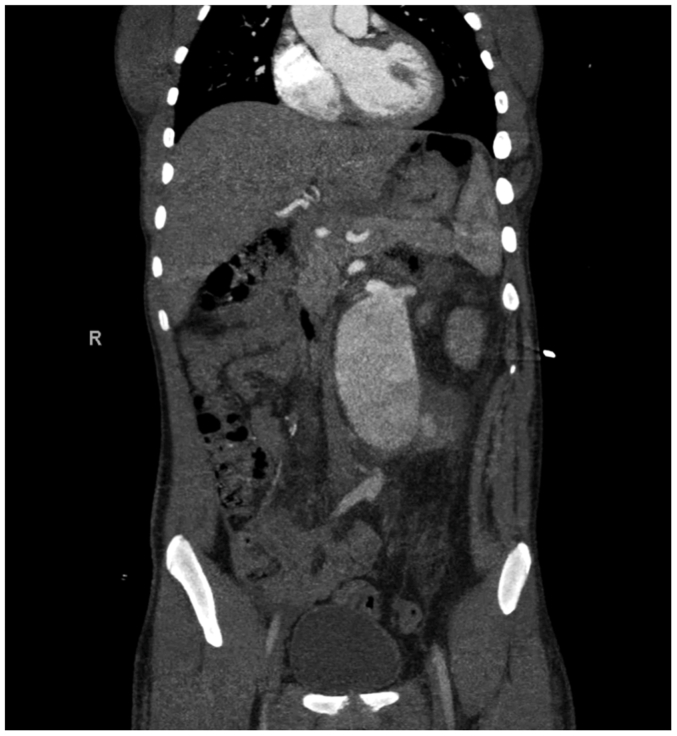
Computed tomography angiography of the ruptured abdominal aortic aneurysm (AAA) with active retroperitoneal extravasation (coronal view).

**Fig 3:**
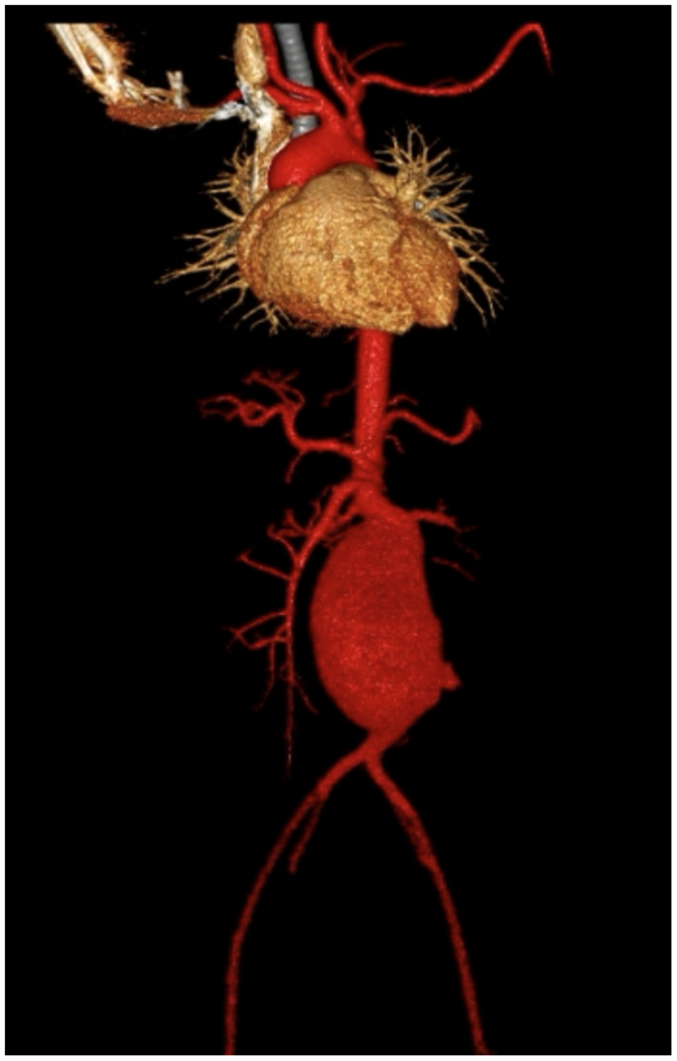
Three-dimensional computed tomography angiographyreconstruction of ruptured infrarenal abdominal aortic aneurysm (I-AAA).

Laparotomy revealed a 7.2-cm purely I-AAA with contained rupture into the retroperitoneum. A transperitoneal approach was undertaken given the left lateral retroperitoneal nature of the rupture. This strategy allowed appropriate aortic control distal to the bilateral renal arteries before aortic transection. Transperitoneal infrarenal aortic and bilateral common iliac artery control were obtained swiftly. The patient was heparinized in the standard fashion. Aortotomy, lumbar artery ligation, and appropriate hemostasis were performed. Notably, the radiographically identified posterior left lateral rupture site was easily confirmed on examination of the aneurysmal aortic wall (Fig 4).

**Fig 4:**
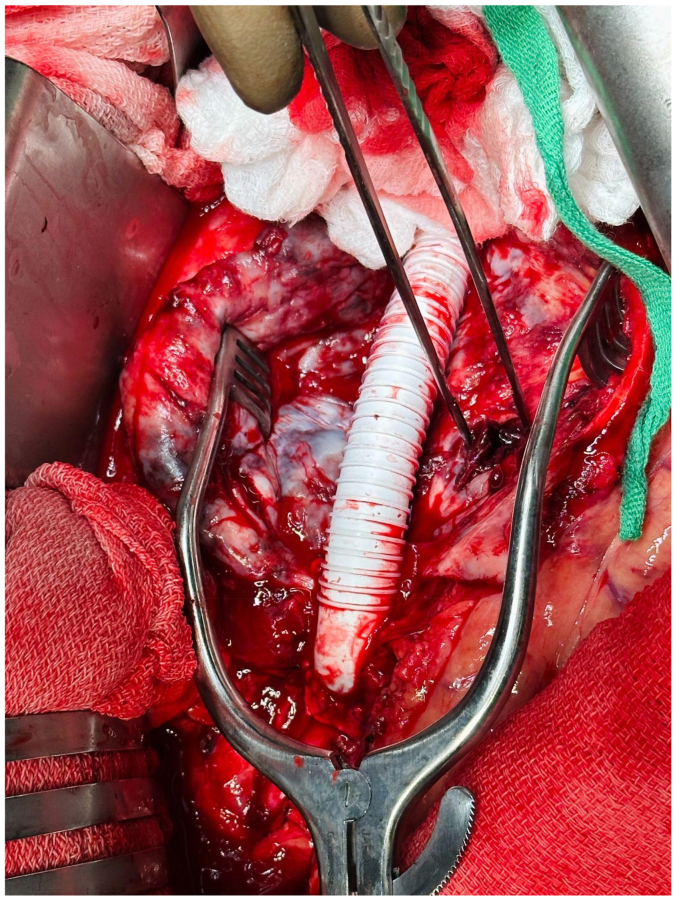
Intraoperative abdominal aortic aneurysm (AAA) repair and polytetrafluorethylene (PTFE) graft reconstruction with rupture site.

The infrarenal to aortic bifurcation was then reconstructed with an 14-mm diameter tubular polytetrafluorethylene (PTFE) graft tailored to an appropriate length for optimal interpositioning. Moreover, we anticipated the need for a long-term and slightly oversized graft option that would be suitable for decades of use. As such, after expert discussion, we elected to use the PTFE tubular graft platform to perform our aortic reconstruction. Although Dacron (Invista) is certainly a reasonable option, we believed that PTFE would afford the patient a more durable repair. The graft was sewn in place and buttressed with felt pledgeting proximally and distally. Graft felt pledgeting was used to reinforce anastomoses because we knew these anastomoses would certainly evolve and potentially degenerate with normal adolescent development. The aortic aneurysm sac was further closed around the graft. The patient was noted to have palpable femoral and pedal pulses before closing the abdomen in the standard fashion. The patient was then admitted to the surgical intensive care unit postoperatively.

The patient was extubated within 24 hours postoperatively and experienced a largely uneventful hospital course, with discharge to the neighboring children's hospital on postoperative day 4. Repeat computed tomography angiography was performed before discharge, which demonstrated an intact I-AAA repair without major surgical concern. She was ultimately discharged home on postoperative day 7. At the 3-month postoperative follow-up visit, she was noted to have recovered well from surgery and planned for 6-month interval imaging. Patient consent for publication was obtained per institution protocol.

## Discussion

First named by Von Recklinghausen in the 19th century, TSC is an autosomal-dominant, variably penetrant syndrome of benign tumors and dysplastic lesions affecting multiple organ systems.^1^^,^^3^ The association of TSC and aortoiliac aneurysms is exceedingly rare. The current literature characterizes TSC-related AAAs as large, rapidly progressive, and often ruptured at the time of presentation.^4^ As a result, there is a limited history of case reports, case series, and expert opinion advocating for routine AAA screening and early operative intervention in all patients with TSC.^1^, ^2^, ^3^, ^4^, ^5^ Although no formal guidelines are established, elective AAA repair is recommended in all patients with TSC.^6^ Early detection and repair can spare the patient the morbidity of various complications or mortality of AAA rupture.

The exact histopathology of developing AAAs in patients with TSC is not clearly defined; however, it has been suggested that abnormalities in connective tissues are a possible cause.^7^ Histopathological analyses of previous abdominal and thoracic aortic aneurysms have yielded a loss of elastin fibers similar to Marfan syndrome.^7^^,^^8^ Aneurysmal degeneration may be related to the inherent weakness in the aortic wall of patients with TSC.

The average 14-year-old girl with TSC will continue to physically and physiologically develop into her early 20s. With the mean life expectancy of a patient with TSC to be 66 years, we expect our patient to reach a similar decade of life.^9^ Moreover, we made a calculated decision to use an appropriately sized (14-mm) PTFE aortic tube graft, given the significantly less graft dilatation at 6 years and equal infection risk to Dacron aortic grafts.^10^, ^11^, ^12^

## Conclusions

We report the successful perioperative outcome of a young girl with TSC presenting with a ruptured I-AAA. Overall, given the limited data, clinicians should maintain a high index of suspicion for aortic lesions in patients with TSC, consider targeted surveillance imaging, and contribute case data to the literature to refine surveillance and treatment strategies for this unique patient population.

## Funding

None.

## Disclosures

None.
